# Human Stem Cell-Derived Astrocytes: Specification and Relevance for Neurological Disorders

**DOI:** 10.1007/s40778-016-0049-1

**Published:** 2016-06-03

**Authors:** Giulia Tyzack, Andras Lakatos, Rickie Patani

**Affiliations:** 1 Department of Molecular Neuroscience, UCL Institute of Neurology, Queen Square, WC1N 3BG London, UK; 2 John van Geest Centre for Brain Repair, Department of Clinical Neurosciences, University of Cambridge, E.D. Adrian building, Forvie site, Robinson way, Cambridge, CB2 0PY UK; 3 Addenbrooke’s Hospital, Cambridge University Hospitals, Hills Rd, Cambridge, CB2 0QQ UK; 4 Department of Clinical Neurosciences, University of Cambridge, Cambridge, UK; 5 Euan MacDonald Centre for MND, University of Edinburgh, Edinburgh, UK

**Keywords:** Astrocytes, Pluripotent stem cells, Disease modelling, Neurodegeneration

## Abstract

Astrocytes abound in the human central nervous system (CNS) and play a multitude of indispensable roles in neuronal homeostasis and regulation of synaptic plasticity. While traditionally considered to be merely ancillary supportive cells, their complex yet fundamental relevance to brain physiology and pathology have only become apparent in recent times. Beyond their myriad canonical functions, previously unrecognised region-specific functional heterogeneity of astrocytes is emerging as an important attribute and challenges the traditional perspective of CNS-wide astrocyte homogeneity. Animal models have undeniably provided crucial insights into astrocyte biology, yet interspecies differences may limit the translational yield of such studies. Indeed, experimental systems aiming to understand the function of human astrocytes in health and disease have been hampered by accessibility to enriched cultures. Human induced pluripotent stem cells (hiPSCs) now offer an unparalleled model system to interrogate the role of astrocytes in neurodegenerative disorders. By virtue of their ability to convey mutations at pathophysiological levels in a human system, hiPSCs may serve as an ideal pre-clinical platform for both resolution of pathogenic mechanisms and drug discovery. Here, we review astrocyte specification from hiPSCs and discuss their role in modelling human neurological diseases.

## Introduction

Astrocytes are specialised cells that are classified as central nervous system (CNS) glia, together with oligodendrocytes and microglial cells. During development, the specification of glia (gliogenesis) follows that of neurons (neurogenesis). In the late embryonic stage and early postnatal period, astrocyte precursors are specified from neural precursor cells (NPCs) via Notch signalling [1]. In a process that is reminiscent of neuronal development, astrocyte precursors migrate away from germinal zones in a radial manner before they differentiate and mature, acquiring unique region-specific functional attributes [2]. Once fully mature, astrocytes occupy largely non-overlapping three-dimensional domains, allowing for functional compartmentalisation within the neuraxis [3]. At the boundaries between domains, gap junctions are formed to facilitate the spread of ions and signalling molecules across the astrocyte network [4]. The complexity of mature astrocytes is structurally exhibited by up to eight major (or ‘stem’) processes, each of which gives rise to hundreds of thousands of fine, elaborated subprocesses or ‘leaflets’ that intimately contact synapses, dendrites and blood vessels [5–8]. Such arborisation is crucially related to the diverse roles played by astrocytes, together with the careful harmonisation and functional coupling of these activities.

Each astrocyte can contact up to a million individual synapses [9]. During development, astrocytes control the formation, maturation and elimination of synapses [10]. In the adult brain, perisynaptic astrocytic processes intimately enwrap dendritic spines and presynaptic terminals forming the tripartite synapse, which in turn allows astrocytes to respond to synaptic activity and regulate synaptic transmission [5, 6, 11]. By bridging connections between neurons, other glial cells and the vasculature, astrocytes control a number of homeostatic processes. These include the supply of glucose and lactate as energy sources for neurons and the regulation of extracellular pH, ion and neurotransmitter concentration throughout the brain [12, 13]. Through contact with blood vessels, astrocytes regulate blood flow in response to the metabolic activity of different brain areas [14]. By virtue of these homeostatic functions, astrocytes strongly reinforce neuronal survival and function.

Although reports on a precise neuron/glia ratio in the brain vary, the consensus view is that in the human brain they exist in equivalent numbers, with astrocytes comprising up to 40 % of all CNS cells [15]. Region-specific differences in this ratio are likely to have functional consequences; for example, astrocytes outnumber neurons in the cortex, which is in contrast to the ratio within the cerebellum. Recognition of profound heterogeneity is important when considering the role of astrocyte structure and function in disease [16].

### Region-Specific Structure and Function of Astrocytes

Classical taxonomy codifies astrocytes into protoplasmic and fibrous subtypes, which are found in the grey and white matter, respectively [17]. While these descriptions retain validity, accumulating data from genetic and lineage-tracing approaches have highlighted substantial astrocyte heterogeneity (both morphological and functional), raising the prospect of taxonomic reclassification to reflect this previously under-recognised diversity [2, 18–20]. Astrocytic heterogeneity beyond protoplasmic and fibrous subtypes is partially reflected in their morphological status including radial glia of the developing brain (later forming the Muller glia of the retina, Bergmann glia of the cerebellum or stellate astrocytes elsewhere), velate astrocytes of the cerebellum, cortical interlaminar astrocytes, tanycytes in periventricular areas, pituocytes in the neurohypophysis and perivascular astrocytes. Additionally, ependymal cells, retinal pigment epithelium and cells constituting the choroid plexi have also been broadly considered as belonging to the astrocyte lineage. In addition to this morphological heterogeneity, astrocytes exhibit considerable functional diversity in their expression of neurotransmitter receptors, ion channels, transporters and other molecules. For example, cortical astrocytes express high levels of GLT-1 and moderate levels of GLAST [21], while spinal cord astrocytes express significantly lower levels of GLT-1 [22] but specifically express glycine receptors. Inward rectifying potassium channels are also differentially expressed in astrocytes in a region-specific fashion, with Kir4.1 being more abundant in the ventral spinal cord compared to the dorsal regions [23]. In the ventral mesencephalon, but not in other parts of the CNS, astrocytes express Wnts that locally regulate the generation of dopaminergic neurons [24] and dopamine receptors. Similarly, the expression of Sema3a by ventral spinal cord astrocytes is required for proper sensorimotor circuit organization and function [25]. Such regionally determined functional heterogeneity is central to understanding the pathogenesis of region-specific neurological disease [26••]. It follows that the generation of region-specific astrocytic populations is crucial to accurately determine their role in disease.

### The Need for a Human Astrocyte Experimental Platform

Accumulating evidence of a multitude of astrocyte-mediated homeostatic processes has challenged traditional ‘neuron-centric’ views of neurological disease. Indeed, several studies have highlighted the relevance of non-cell autonomous astrocyte-dependent mechanisms in neurodegenerative disorders [27]. In recent years, animal models have provided fundamental insights into the role of astrocytes in neurodegeneration. Notwithstanding the undeniable utility of animal models, complementary platforms to investigate astrocyte biology in human systems are necessary in order to more precisely capture clinical pathophysiology by avoiding potential interspecies differences. Indeed, an increasing body of evidence suggests that significant differences exist between human and rodent astrocytes. Human astrocytes have a more complex structure than their rodent counterparts, occupying an almost 30-fold larger volume, and extending 10-fold more processes [28, 29]. This structural complexity is also reflected in functional properties, which are not shared by astrocytes of other species. For example, mature human astrocytes propagate calcium waves more rapidly than their rodent counterparts [29, 30, 31••] and show a more robust response to glutamate [31••, 32], consistent with an increased capacity to sense and respond to synaptic activity. Chimeric mice receiving transplants of human astrocyte precursors show increased levels of excitatory synaptic transmission, enhanced long-term potentiation (LTP) and improved learning and memory [30]. The difference between human and rodent astrocytes is also reflected in their transcriptome, with over 600 genes being enriched in human but not mouse astrocytes. Among these differentially expressed genes, common themes emerge including divergent calcium handling properties, as alluded to above [31••]. In view of the limited accessibility to human non-transformed astrocytes for disease modelling, patient-specific human induced pluripotent stem cells (hiPSCs) represent an attractive strategy to derive highly enriched astrocytic populations for further study.

### Specification of Astrocytes from hiPSCs: A Developmental Perspective

**Fig. 1:**
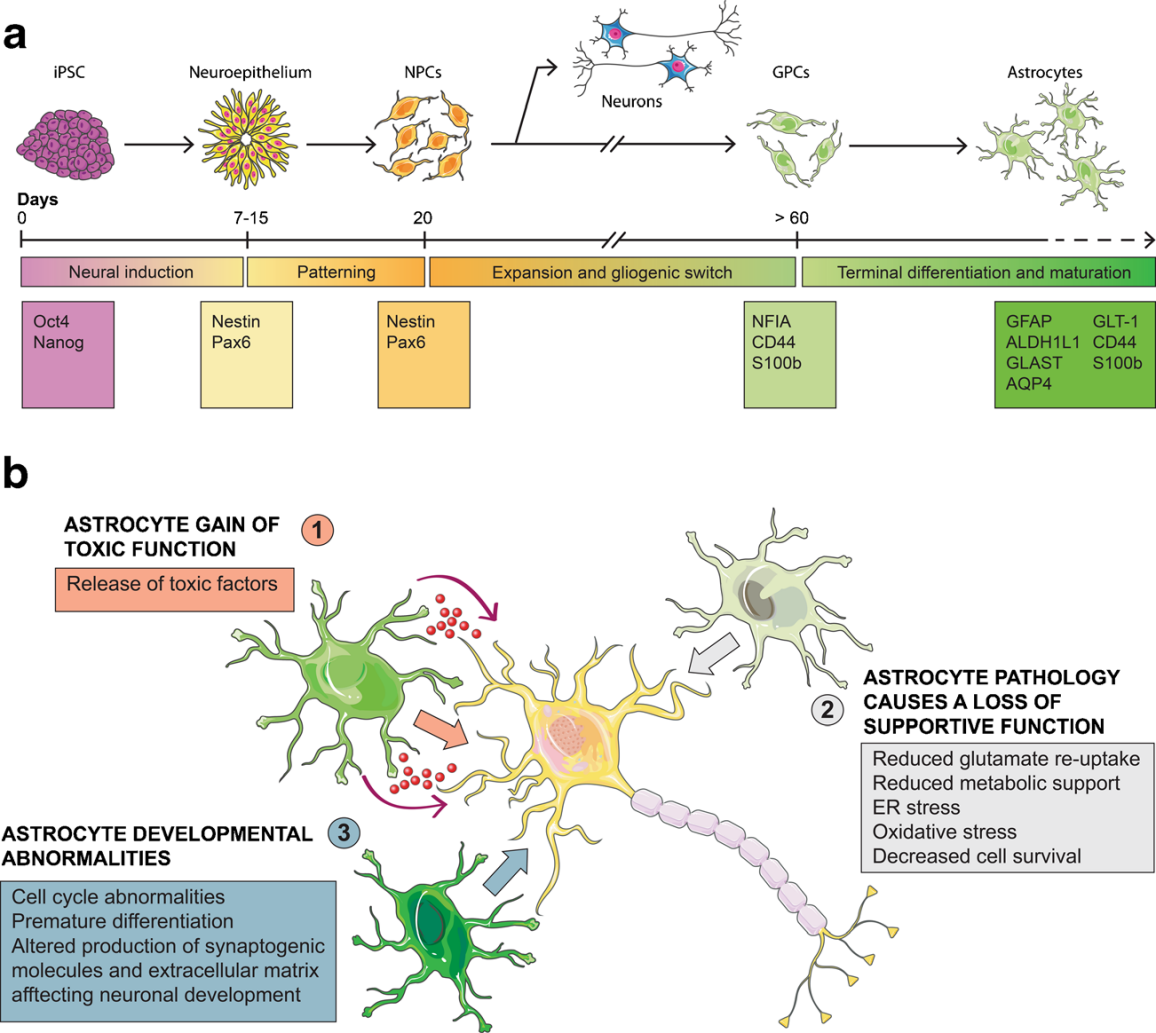
Modelling neurodegeneration using hiPSC-derived astrocytes. **a** Diagram of directed differentiation of astrocytes from hiPSCs. HiPSCs are initially converted into rosette-forming neuroepithelial cells. After neural conversion, morphogens can be added for regional patterning of NPCs. NPCs can be subsequently expanded either in adhesion or in suspension in presence of growth factors. Early neurogenic progenitors will spontaneously differentiate into neurons upon growth factor withdrawal. To generate astrocytes, long-term expansion (>60 days) of NPCs is required to allow the gliogenic switch to occur. Terminal differentiation may be accelerated using morphogens or epigenetic modulators. Markers for the different stages of differentiation are listed in the corresponding boxes. **b** Schematic representation of the possible involvement of astrocytes in neurologic conditions. Diseased astrocyte can directly be harmful to neurons via the release of toxic factors such as inflammatory mediators and ROS (*1*). Alternatively, an astrocyte cell-autonomous pathology could impair their homeostatic and trophic functions, resulting in neuronal damage due to lack of support (*2*). Lastly, an intrinsic abnormality of astrocyte development could alter neuronal maturation and function (*3*). Diagrams were drawn using templates freely available from Servier Medical Art (http://www.servier.co.uk/content/servier-medical-art)

Astrocytes are differentiated from hiPSCs using ontogeny-recapitulating methods, in a similar fashion to their region-specific neuronal counterparts. It is noteworthy that astrocyte development is comparatively understudied and poorly defined, partially due to the lack of reliable markers [33–35]. Protocols for generating astrocytes from hiPSC are broadly operationalised in four main phases: (1) the neural induction phase, when hiPSCs lose their pluripotency and are converted into NPCs; (2) neural patterning to positionally specify astrocytes to defined regions of the CNS; (3) the gliogenic switch, which marks the transition of NPCs from neurogenic to gliogenic—a temporally determined, cell intrinsic and likely epigenetically mediated process and (4) astrocyte terminal differentiation, during which gliogenic precursors terminally differentiate into astrocytes (Fig. 1a). These phases are discussed in further detail hereunder.

#### Neural Induction

HiPSCs are first converted into multipotent neuroepithelial cells in a process defined as neural induction. The more robust protocols for neural induction rely on dual inhibition of SMAD signalling in adherent hiPSC cultures [36]. These protocols eliminate the use of stromal feeders and avoid the formation of embryoid bodies [37, 38], providing a reliable platform to achieve efficient neural conversion in chemically defined medium. During neural conversion, the downregulation of pluripotency genes, such as Nanog and Oct4, is mirrored by the acquisition of neural stem cell markers including Pax6 and nestin [36]. In parallel, a morphological transition from hiPSCs with large nuclei to a tightly packed neuroepithelial sheet with notably smaller nuclei is observed. Neuroepithelial differentiation can also be confirmed by the appearance of neural rosettes [36]. The newly generated NPCs can be maintained either in adherent monolayer or suspension culture, and they can be expanded in vitro in the presence of growth factors such as fibroblast growth factor 2 (FGF-2) or epidermal growth factor (EGF) [39].

#### ‘Patterning’ Strategies to Generate Region-Specific Astrocytes

A wealth of region-specific functional neuronal subtypes have been specified from hiPSCs (reviewed in [40, 41]), although certain cell fates remain challenging, including cerebellar derivatives [42]. Comparatively less experimental attention has been directed towards glia. Recent evidence suggests that morphological and functional astrocyte diversity is determined during development by regional patterning of neural precursors, thus allowing similar developmental principles to be applied for their specification from hiPSCs [18, 35, 43]. In vitro, the default regional identity acquired by hiPSC-derived NPCs is a telencephalic [44]. After neural conversion, newly generated NPCs can be patterned along rostro-caudal and dorso-ventral axes using extrinsic morphogenetic instruction. The administration of FGF and retinoic acid (RA) determines rostro-caudal identity [43, 45]. Wnts, bone morphogenetic proteins (BMPs) and sonic hedgehog (Shh) are utilised to specify NPCs along the dorso-ventral axis [44], although the actual effects of each morphogen are more complex and context-dependent. It was recently shown that the positional identity acquired during patterning is retained at later stages of astroglial specification—described below—as confirmed by expression of homeodomain-specific transcription factors [35, 43, 46, 47]. This approach therefore provides a powerful platform to generate region-specific astrocytes. Further experimentation is required to realise the full morphological and phenotypic diversity of astrocytes in the CNS. The development of in vivo visualisation tools and high-throughput transcriptomic approaches raise the prospect of a more integrated morphological and functional classification that captures the true complexity of astrocyte heterogeneity [48].

#### The Gliogenic Switch

Neurogenesis precedes gliogenesis in vivo, with precursors switching developmental programmes from the production of neurons to the generation of glial cells. This process, known as the gliogenic switch, depends on two related events: the inhibition of neurogenesis and the activation of gliogenesis. Understanding the mechanisms underlying the gliogenic switch is crucial to define optimal strategies for differentiating astrocytes from hiPSC-derived NPCs. A key element with dual function in the gliogenic switch is Notch signalling [49]: on the one hand, Notch inhibits proneural basic helix loop helix (bHLH) factors via HES proteins. On the other hand, it promotes astrogliogenesis by activating the JAK-STAT pathway [50], a key regulator of astrocyte development [51]. Together with Notch, several cytokines secreted by differentiating neurons also converge on the JAK-STAT pathway, thus promoting astrocyte specification; these include members of the interleukin 6 (IL-6) family, such as leukaemia inhibitory factor (LIF), ciliary neurotrophic factor (CNTF) and cardiotrophin 1 (CT1) [52]. BMPs also synergistically interact with these cytokines, inducing the formation of a Smad1-STAT3 complex that transactivates astrocyte-associated genes [53], while also inhibiting proneural bHLH transcription factors [54]. Together with Notch, nuclear factor I (NFI) genes, and in particular NFIA, have an instructive role in promoting astrogliogenesis, by directly regulating the expression of the astrocyte genes GFAP [55] and GLAST [56]. For NPCs to acquire gliogenic competence, key epigenetic changes occur including a wave of de-methylation of astrocyte-specific genes, which regulates the switch from early (neurogenic) to late (gliogenic) precursors in the developing rodent brain [51, 57, 58]. Accordingly, global DNA hypomethylation leads to the activation of the JAK-STAT pathway and induces precocious astrogliogenesis, both in vitro and in vivo [59].

Recapitulating these developmental milestones, NPCs derived from hiPSCs initially have high neurogenic potential and limited capability to generate glial cells [43], possibly due to hypermethylation of astrocyte genes [60]. Indeed, early NPCs (2–4 weeks of differentiation in vitro) would spontaneously differentiate into neurons upon growth factor withdrawal [36, 61]. However, after long-term expansion in vitro, in the presence of FGF and/or EGF, NPCs spontaneously undergo the gliogenic switch. This results in a progressive reduction of their neurogenic potential, paralleled by an increased competence to generate astrocytes. Under these conditions, and by around 12–15 weeks, the majority of precursors are positive for the immature astrocyte markers NFIA, S100β and CD44 [35, 39, 43]. While remaining proliferative, these cells express the glutamate transporter GLT-1 (known as EAAT-2 in humans), possess potassium currents and can induce synaptogenesis—all features consistent with an immature astrocytic phenotype [43]. At this stage, immature astrocytes are also responsive to signals inducing their terminal differentiation and maturation.

#### Promoting Terminal Differentiation

Immature proliferative astrocytes can be induced to spontaneously differentiate by removing mitogens from the culture medium [39]. Despite generating a highly enriched astrocyte culture, this method requires protracted culture durations (>120–180 days). For this reason, developmental insights have been exploited to accelerate functional maturation of gliogenic precursors/immature astrocytes. Such strategies include the use of: (1) interleukins of the IL-6 family such as CNTF [43, 62, 63••] and LIF [64]; (2) BMPs, alone or in combination with the aforementioned cytokines [64, 65] and (3) neuregulin, which also activates the JAK-STAT pathway [65]. Others have reported the use of serum to induce efficient terminal differentiation of astrocytes [46, 66]. However, serum is known to induce irreversible reactive changes in cultured astrocytes [19, 31••, 67]. Interestingly, and in contrast to other studies showing that FGF-2 maintains immature astrocytes in a proliferative state, one report demonstrated that FGF-1 and FGF-2 can induce the differentiation of mature quiescent astrocytes with low GFAP expression but high levels of the glutamate transporter GLT-1 [46]. Lastly, based on the epigenetic changes that accompany the gliogenic switch in vivo [51], modifiers such as the DNA methyltransferase inhibitor Aza-Cytidine and the histone deacetylase inhibitor Trichostatin-A have been used to accelerate astrocyte differentiation from NPCs [60].

### Characterisation of hiPSC-Derived Astrocytes

One of the most pressing issues regarding the characterisation of hiPSC-derived astrocytes is the lack of reliable markers defining different astrocytic subtypes and their stage of maturation. Generally, precursors committed to the astrocyte lineage and immature astrocytes are characterised by the expression of the transcription factor NFIA [55, 56]. S100β has also been widely used as an astrocyte progenitor marker; however, it is also expressed in oligodendrocyte precursors/NG2 cells [68]. For some time, GFAP has been considered a gold standard marker for mature astrocytes; however, its expression varies dramatically depending on regional identity, activation state and ageing [69]. Therefore, in parallel to GFAP, other markers should be considered including ALDH1L1, CD44, GLT-1, GLAST, Acquaporin4 and Connexin43 [26••, 31••, 70].

In addition to assessing the expression of specific markers, several assays are available to characterise the functional properties of hiPSC-derived astrocytes. It is therefore possible to assess whether patient-specific hiPSC-derived astrocytes show any intrinsic defect or cell autonomous pathology, thereby recapitulating a specific disease-related phenotype. This can be carried out by morphological analyses, examining the number, volume and length of astrocytic processes [31••, 71••]. Astrocytes can also be tested functionally by measuring their electrophysiological properties, glutamate uptake, calcium wave propagation upon mechanical stimulation and calcium signalling in response to ATP and glutamate [26••, 31••, 43, 63••, 72•]. On the other hand, non-cell autonomous effects of hiPSC-derived astrocytes on maturation, function and survival of neurons can be evaluated using co-culture or astrocyte conditioned media paradigms. A classical assay makes it possible to assess immature astrocytes’ ability to induce synapse formation and maturation, using co-culture with retinal ganglion cells, mouse or human neurons [31••, 63••, 72•]. Co-culture also allows evaluation of the astrocyte’s impact on neuronal viability, providing a simplified in vitro system to investigate signalling pathways involved in a neuroprotective or toxic effect of these cells [31••, 73•]. Lastly, to analyse cell-cell interactions in a more complex environment, hiPSC-derived astrocytes can be transplanted into rodent models, assessing their integration and function in vivo [43, 65]. The approaches described above allow the generation of highly enriched astrocyte cultures that represent a powerful tool to investigate developmental and disease-related molecular mechanisms. For experimental use, it is important to consider both the state of astrocytic maturation and reactivity as these may influence experimental outcomes.

### Astrocyte Maturation

Astrocytes exist in two distinct maturational stages: (1) foetal, during which they are immature and proliferative and (2) a postnatal phase when astrocytes exit the cell cycle and terminally differentiate [31••, 46, 70, 74]. Astrocytes maximally express markers of maturity after 6–12 months of age in humans [31••]. As astrocytes mature, their gene expression profile changes dramatically with an upregulation of genes involved in synaptogenesis, cell-cell signalling, fatty acid metabolism, cell adhesion and ion homeostasis [31••], suggesting that they exhibit differential functional competencies that are determined by maturational state. Importantly, the temporal coincidence of astrocytic maturity with synaptogenesis underlies their pivotal role in orchestrating the formation of neuronal connections, not just during development but for the dynamic synaptic remodelling that occurs throughout adult life. When astrocytes are generated in vitro from hiPSCs, they are likely to reflect a foetal maturational state, as already demonstrated for their hiPSC-derived neuronal counterparts [75–77]. Each hiPSC-derived astrocyte culture is also likely comprised by a mixture of cells at different stages of differentiation, ranging from precursors to mature astrocytes [26••]. Additionally, pure astrocytic cultures lack instructive developmental signals from other neural cells that would otherwise be present in vivo, which might affect the expression of astrocyte-specific proteins [78]. In light of the significant functional differences between immature and mature astrocytes in physiological and pathological conditions, the appropriate experimental paradigm should be carefully considered to capture specific and experimentally relevant phenotypes.

### Astrocyte Reactivity

Astrocytes in culture often resemble those responding to CNS injury and are collectively termed ‘reactive astrocytes’. This state encompasses a number of attributes including astrocyte proliferation, hypertrophy, structural and functional remodelling of their processes. For many decades, reactive astrocytes have been regarded as detrimental for repair; however, recently, it is increasingly recognised that they also choreograph neurorestorative processes [69, 79]. These responses are complex and dynamic over each disease course, and can include varying degrees of the following injury-specific effector mechanisms: immunoregulation, functional lesion isolation, neurovascular reconfiguration and adaptation of neuronal connectivity [80•]. It transpires that the specific repertoire of morphological and functional changes that accompany astrocytic reactions is determined by the extent, nature and anatomical location of the injury. At the lesion site, robust astroglial structural and functional changes occur, leading to disruption of domain architecture and accumulation of inflammatory cells, precursors and meningeal fibroblasts, which conspire to form the glial scar (this process is termed ‘anisomorphic astrogliosis’). This is associated with production of substances that may inhibit axonal regeneration and neurite growth, including chondroitin sulphate proteoglycans. Conversely, astrocytic response in mild insults, or remotely from the injury site, causes far less structural change (termed ‘isomorphic astrogliosis’). Despite a less conspicuous morphological cellular change, these astrocytes exhibit more favourable functional changes, which are associated with neuroprotection, axon repair and synaptic plasticity [81, 82]. Against this background, and depending on the experimental paradigm, either resting or reactive astrocytes (or both) may be required for specific assays. Most of the soluble factors used to accelerate astrocyte differentiation in vitro, such as cytokines and BMPs, are also effective inducers of astrocyte reactivity and can induce irreversible alterations of astrocytic function [83]. While reactive astrocytes might recapitulate the response to a specific pathological condition, prolonged exposure to activating stimuli could alter or even mask disease phenotypes [26••]. If the experimental paradigm requires a more physiological model of accelerated astrocyte maturation in the absence of activation, a recent report suggests the use of FGF-1 to induce a quiescent mature phenotype [46]. In neurological disease, however, astrocytes predominantly show some form of reactive transformation in affected areas, therefore reverting them into a quiescent phenotype in vitro may actually impede modelling neurodegeneration with fidelity and precision.

### Modelling Neurodegeneration

Noting the complexity of astrocyte-mediated homeostatic functions, their implication in a number of CNS disorders is somewhat unsurprising. Whether astrocyte contribution to disease onset and progression is due to a loss of their supportive and homeostatic function, or to a gain of toxic functions—or a combination of both—remains unresolved in the majority of neurodegenerative conditions (Fig. 1b) [27]. The use of patient-specific hiPSC-derived astrocytes therefore represents a powerful tool to address this question, by recapitulating initiating pathogenic events against a human genetic background, where mutations are conveyed at pathophysiological levels. Here, we review recent progress in the field with a specific focus on studies using hiPSC-derived astrocytes to interrogate their role in neurodegeneration (further summarised in Table 1).

**Table 1:** Summary of studies using hiPSC-derived astrocyte to investigate disease mechanisms

Disease	Mutated gene(s)	Culture method	Astrocyte phenotype	Effect on neurons	Ref.
AD	APP	Neural conversion with formation of embryoid bodies. Expansion of NPCs in presence of BDNF and GDNF. Terminal differentiation induced by 10 % serum	Intracellular accumulation of Aβ, increased ROS production, ER stress	N/A	[84]
ALS	SOD1, C9orf72, sporadic cases	Direct reprogramming of patient fibroblast to iNPCs. Terminal differentiation induced by 10 % serum	No astrocyte pathology observed	Reduced neurite outgrowth and neuronal survival in co-cultures	[73•]
	TARDBP	iPSC-derived NPCs expansion in suspension in presence of EGF and FGF-2 Terminal differentiation induced by CNTF	Cytoplasmic accumulation of TDP-43, reduced astrocyte survival	No toxicity	[63••]
CS	HRAS	iPSC-derived NPCs expansion in suspension in presence of EGF and FGF-2 Terminal differentiation induced by CNTF. Astrocytes analysed after 7 days of terminal differentiation	Accelerated astrocyte maturation, hyperplasia, increased release of proteoglycans and extracellular matrix components	In co-culture, premature maturation of neurons, increased neurite outgrowth and increased synaptic density	[71••]
DS	Trisomy of Chr 21	iPSC-derived NPCs expansion in suspension in presence of FGF-2. Terminal differentiation induced by FGF2 and BMP4	Enhanced astrocyte differentiation. Higher expression of GFAP and S100β, increased production of ROS, decreased expression of synaptogenic molecules	In co-culture, reduced neuronal survival, reduced ion channel maturation and synapse formation	[72•]
HD	Htt	iPSC-derived NPCs expansion in suspension in presence of FGF-2. Terminal differentiation induced by 2 % serum	Astrocyte vacuolation	N/A	[85]
RS	MECP2	iPSC-derived NPCs expansion in suspension in presence of FGF-2. Terminal differentiation induced by growth factors withdrawal	Enhanced astrocyte differentiation. Higher expression of GFAP and S100β.	N/A	[86]
	MECP2	iPSC-derived NPCs expansion in adhesion in presence of FGF-2. Terminal differentiation induced by CNTF	Altered microtubule dynamics. Impaired vesicular transport	N/A	[87]
	MECP2	iPSC-derived NPCs expansion in suspension in presence of EGF and FGF-2. Terminal differentiation induced by CNTF	No phenotype described.	In co-culture, mouse hippocampal neurons show reduced neurite outgrowth and reduced frequency of postsynaptic currents	[88]
SMA	SMN1	iPSC-derived NPCs expansion in suspension in presence of EGF and FGF-2. Terminal differentiation induced by CNTF	Increased GFAP expression, decreased process length, impaired calcium signalling	N/A	[89]
	SMN1	iPSC-derived NPCs expansion in suspension in presence of EGF and FGF-2. Terminal differentiation induced by growth factor withdrawal	No alteration of mitochondrial bioenergetics and oxidative stress markers	N/A	[90]

#### Amyotrophic Lateral Sclerosis (ALS)

ALS is a progressive neurodegenerative disease characterised by selective degeneration of both upper and lower motor neurons (recently reviewed in [91]). Early astroglial atrophy is reported in the immediate vicinity of spinal motor neurons—prior to their degeneration—in animal models of SOD1-related ALS [92]. Indeed, since the observation that loss of the astrocytic glutamate transporter GLT-1 leads to motor neuron death by excitotoxicity [93, 94], converging lines of evidence have demonstrated that astrocytes contribute to disease progression in a non-cell autonomous manner [95]. Using hiPSC-derived astrocytes from patients with familial and sporadic ALS, some studies have attempted to elucidate non-cell autonomous pathogenic mechanisms. In one study, astrocytes were directly reprogrammed from ALS patients carrying *C9ORF72* hexanucleotide expansion, mutations in *SOD1* and sporadic cases. ALS-astrocytes, either from familial or sporadic cases, were found to be deleterious to both motor neuron survival and neurite outgrowth in co-culture paradigms. To determine whether this effect was dependent on ALS-astrocyte toxicity versus lack of support, co-cultures were supplemented with wild-type astrocyte conditioned medium. This approach failed to rescue motor neuron cell death, suggesting a toxic gain of astrocytic function [73•]. HiPSC-derived astrocytes from patients carrying *TARDBP* mutations show abnormalities typical of a TDP-43 proteinopathy, including its cytoplasmic mislocalisation. Longitudinal imaging of mutant astrocytes revealed that TDP-43 mislocalisation decreases cell survival, suggesting that mutant TDP-43 is responsible for astrocyte pathology. In this case, however, when co-cultured with either control or mutant TARDBP motor neurons, mutant astrocytes were not toxic [63••]. This result is in apparent contrast with the non-cell autonomous toxicity previously reported in the context of sporadic, C9ORF72 and SOD1 mutations [73•, 96–98]. However, these findings can be reconciled through the possibility of mutation-specific astrocyte pathology in familial ALS, therefore suggesting at least partially divergent disease mechanisms in astrocytes. Further systematic astrocyte-neuron interaction studies are essential to precisely elucidate key aspects of cellular autonomy in vitro using functional and high-throughput molecular assays in hiPSC systems.

#### Alzheimer’s Disease (AD)

AD is the most common cause of dementia and is characterised by a progressive decline in cognitive functions, especially episodic memory. Histopathologically AD brains show characteristic deposition of intra-neuronal neurofibrillary tangles and extracellular β-amyloid (Aβ) plaques. Reactive astrocytes are found in association with Aβ plaques, but their contribution to disease progression is still unclear [99]. Astrocytes can internalise and degrade extracellular Aβ via ApoE [100, 101]. However, the intracellular accumulation of Aβ in mouse astrocytes results in abnormal calcium influx and glutathione depletion. This reduction of the antioxidant defence in astrocytes results in impaired neuronal viability after exposure to Aβ oligomers, suggesting that neuronal cell death in this model is a consequence of impaired astrocytic ability to support neuronal survival [102]. Additionally, a recent study describes cell-autonomous pathology in both hiPSC-derived neurons and astrocytes from patients with either familial or sporadic AD. AD astrocytes showed intracellular accumulation of Aβ, increased ER stress and ROS production. However, the effect of AD astrocytes on neuronal function and survival was not directly examined [84].

#### Parkinson’s Disease (PD)

PD is a neurodegenerative disease that presents with both motor and non-motor phenomena. Motor hallmarks include asymmetrical slowing of movements (bradykinesia), rigidity, tremor and postural instability. Although the neuropathological manifestations can be extensive, motor perturbations are anatomically localised to the substantia nigra and more specifically to dopaminergic neurons. The pathological hallmarks of PD include Lewy bodies, which are composed of α-synuclein. Neuron to astrocyte transfer of α-synuclein has been demonstrated along with evidence of astrocyte-related non-cell autonomous mechanisms of injury [103]. Conversely, astrocyte-specific overexpression of Nrf2 and DJ-1 (regulators of protective responses against cellular/mitochondrial oxidative stress) ameliorate cellular phenotypes [104, 105]. Taken together, these facts demonstrate dynamic and crucial roles for astrocytes in PD. The astrocyte to neuron ratio for dopaminergic neurons in the substantia nigra has been suggested to be lower than any other region within the neuraxis [106], raising the hypothesis that these neurons are more vulnerable to perturbed glial support. Systematic studies have yet to comprehensively address the role(s) of region-specific astrocytes in PD and this is an important focus for future hiPSC-based studies.

#### Huntington’s Disease (HD)

HD is a rare neurodegenerative disorder caused by the expansion of a CAG repeat in the huntingtin (*HTT*) gene. Despite having been historically considered a strictly neuronal pathology, recent studies outline a key role for astrocytes in HD pathogenesis. Expression of Htt with expanded CAG repeats in mouse astrocytes manifest a functional atrophy as demonstrated by impaired glutamate transport potentially leading to excitotoxicity [107]. More recently it was shown that, in an HD mouse model, mutant Htt causes the downregulation of a potassium channel in astrocytes in the striatum, thus impairing their ability to buffer extracellular potassium and increasing the excitability of spiny neurons [108]. Whether these astrocytic phenotypes described in rodent models are also shared by human astrocytes remains unknown. To date, only one study has investigated the effects of mutant Htt in hiPSC-derived astrocytes from HD patients. This study reports extensive astrocyte vacuolation that increases with time in culture and correlates with the length of CAG expansion [85]. However, molecular mechanisms or functional implications of this astrocytic phenotype have not been addressed.

#### Modelling Neurodevelopmental Disorders

In light of the instructive role of astrocytes during CNS development, emerging evidence shows that these cells play a crucial role in a number of neurodevelopmental disorders, such as Costello syndrome (CS), Rett syndrome (RS) and Down syndrome (DS) [7]. Patient-specific iPSC-derived astrocytes manifest developmental abnormalities that fundamentally affect the morphology and function of neurons in a non-cell autonomous manner. A common feature reported in these studies is increased differentiation of NPCs towards the astroglial lineage. Possibly the most striking example comes from astrocytes generated from iPSC from patients with CS [71••]. Compared to controls, these astrocytes showed a markedly increased proliferation and accelerated maturation, more complex morphology and hypertrophy. From a functional perspective, a major phenotype of these astrocytes is increased deposition of extracellular matrix remodelling factors and proteoglycans. This in turn culminates in premature maturation of neurons in co-culture, and early formation of perineuronal nets upon transplantation into mouse models of CS [71••]. Similarly, hiPSC-derived NPCs from RS patients showed enhanced astrocytic differentiation with increased expression of GFAP and S100β [86]. Concurrently, altered microtubule dynamics and impaired vesicular transport were identified, suggestive of an intrinsic RS astrocyte dysfunction [87]. In co-culture with mouse hippocampal neurons, RS astrocytes have a significant non-cell autonomous effect on neurons, both morphologically and functionally. HiPSC-derived astrocytes from RS patients cause an impairment in neurite outgrowth and soma size, with significant reduction in the frequency of postsynaptic currents [88]. HiPSC-derived NPCs from DS patients also exhibit increased generation of astrocytes under spontaneous differentiation conditions, with a reciprocal decrease in neurogenesis. Due to reduced production of synaptogenic molecules, DS astrocytes fail to induce full electrophysiological maturation and synapse formation of DS neurons. In addition, DS astrocytes also manifest increased ROS production and lead to diminished survival of DS neurons [72•]. Therefore, the hiPSC system can yield valuable pathomechanistic information underlying neurodevelopmental disorders.

## Conclusions

At present, strategies for astrogliogenesis from hPSCs are protracted and do not fully account for region-specific heterogeneity. Given the complex and diverse roles of astrocytes in neurological disorders, greater experimental attention should be paid to generating regionally defined subtypes of functionally mature astrocytes for further study. A number of important experiments using human systems have uncovered astrocyte cell autonomous or non-cell autonomous mechanisms of disease using neuron-astrocyte co-culture and astrocyte conditioned media paradigms. It follows that astrocytes are emerging as central players in neurodegeneration and their systematic interrogation in conditions traditionally regarded from a neuron-centric perspective is certainly warranted. Given the intimate relationship of astrocytes with neuronal synapses together with their abundance in the CNS, it is plausible that they may provide an alternative cellular target for mechanistically rationalised therapies. Recognising that each model system has limitations, it is crucial to integrate human in vitro systems with animal in vivo models and human post mortem tissue to fully capture the complexity of human neurological disorders. ‘Triangulating’ findings from this integrated approach will in turn lead to high confidence data, which can collectively overcome the limitations inherent in each model system when employed in isolation. We feel strongly that human experimental systems, such as hiPSCs, are key to driving the necessary step change required to discover initiating molecular pathogenic events in neurodegeneration, which in turn will guide the development of desperately needed mechanism-targeting therapies in this discipline.
